# Utilization of Sintered Sludge Ash with Different Mechanical–Thermal Activation Parameters as a Supplementary Cementitious Material: Mechanical Properties and Life Cycle Assessment of Cement-Based Paste

**DOI:** 10.3390/ma17164101

**Published:** 2024-08-19

**Authors:** Tong Lv, Jinrui Zhang, Maoxi Zhao, Jiapeng Yang, Dongshuai Hou, Biqin Dong

**Affiliations:** 1 State Key Laboratory of Hydraulic Engineering Intelligent Construction and Operation, Tianjin University, Tianjin 300072, China; lt_1998@tju.edu.cn (T.L.); 18382171633@163.com (M.Z.); aatroxy_2023@tju.edu.cn (J.Y.); 2 Department of Civil Engineering, Qingdao University of Technology, Qingdao 266000, China; dshou@outlook.com; 3 Guangdong Province Key Laboratory of Durability for Marine Civil Engineering, Shenzhen University, Shenzhen 518060, China; incise@szu.edu.cn

**Keywords:** cement-based paste, life cycle assessment, mechanical properties, mechanical–thermal activation, sintered sludge ash

## Abstract

The proposal of sintered sludge cement (SSC) paste aligns with the low-carbon development goals of building materials. However, there is a lack of scientific guidance for the preparation of sintered sludge ash (SSA). Herein, this study systematically investigates the influence mechanism of mechanical–thermal activation parameters of SSA on the mechanical properties and life cycle assessment (LCA) of SSC paste, and conducts a comprehensive evaluation using a radar chart and the TOPSIS method. The results show that with the increase in calcination temperature and duration, the compressive and flexural strengths of the SSC paste are improved, especially at 600 °C and above, increasing by 57.92% and 62.52%, respectively. The longer calcination time at 1000 °C results in a decrease in its mechanical properties. The addition of SSA significantly reduces the LCA indicators of cement paste. Specifically, 30% SSA only contributes 8.1% to the global warming potential. Compared to calcination, the LCA indicators have less sensitivity to ball milling, and prolonging the time hardly increases them. Based on performance and environmental impact, the optimal SSA is obtained by calcining at 800 °C for 2 h and ball milling for 10 min. This study can provide theoretical guidance for efficient building material utilization of dredged sludge.

## 1. Introduction

China first proposed in 2020 to achieve the goals of carbon peak before 2030 and carbon neutrality before 2060. According to statistics, China’s cement industry emits over 1.3 billion tons of carbon dioxide, accounting for 13% of the country’s total, which is the third largest carbon-emitting industry after the power and steel industries [1]. The main source of carbon emissions is the preparation process of cement clinker through “two grinding and one burning” [2]. As a result, adopting new low-carbon cementitious materials to reduce the use of cement clinker is an effective measure to comply with the dual carbon background [3]. Extensive practice has proven that supplementary cementitious materials (SCMs) mainly composed of industrial solid waste with pozzolanic activity have played a positive role [4], and the joint use of different SCMs, such as fly ash and metakaolin [5], fly ash and nanosilica [6], lithium slag and steel slag, can synergistically improve cement performance [7]. However, the traditional SCMs mentioned above are often generated by heavy industries with high-energy consumption, which will inevitably be replaced by clean industries in the future [8,9].

To maintain the normal ecological environment and the important functions of river and lake systems, China produces over 6 billion m^3^ of sludge annually due to dredging and excavation [10]. As a type of cohesive soil with high moisture content and high porosity, sludge has poor natural mechanical properties and a long consolidation process when buried, resulting in a large amount of land occupation [11]. Meanwhile, sludge contains heavy metal ions and organic waste, posing a risk of secondary pollution, making its resource utilization an inevitable trend [12,13,14]. Compared with traditional utilization methods mainly based on land use and composting, the building material utilization of sludge not only reduces the consumption of natural minerals but also alleviates the pressure on ecological pollution control. Among these methods, using sintered sludge ash (SSA) as a new supplementary cementitious material can effectively achieve the coordinated development of low-carbon building materials industry and sustainable sludge treatment [15,16,17]. The theoretical basis lies in the fact that the main components of sludge are stable crystals represented by quartz and feldspar, as well as clay minerals represented by kaolinite, clinopyroxene, and illite [18,19]. Research has shown that during high-temperature sintering, clay phase minerals undergo dehydroxylation, and their structure changes from ordered to disordered, resulting in changes in the contact state, particle size, and bonding form of sludge particles [20,21]. Through activation treatment, the content of amorphous aluminosilicate in the sludge is increased, which can significantly enhance the pozzolanic activity, allowing it to react with calcium hydroxide in the alkaline environment formed by cement hydration, thereby playing a positive role in the setting, hardening, and performance improvement of cement-based materials [22,23]. Based on our research experience, a higher water–binder ratio is necessary for an effective mixing process due to the loose and porous nature of SSA, and the high-temperature decomposition of polluting organic matter ensures the safety of SSA. However, the selection of specific process parameters for activation treatment methods such as calcination and ball milling in current related research is relatively crude, with most studies directly using parameters for clay and similar raw materials, lacking objective experimental results and scientific analysis to guide the selection of sludge activation processes.

Although researchers have proposed various ways to utilize sludge as building materials, it remains debatable whether the related technology only shifts the pollution stage and whether it is worth being widely promoted. The life cycle assessment (LCA) method, as a tool for statistical calculation of various environmental pollution and emissions generated by products from raw material extraction to final disposal, can effectively quantify various emission indicators and objectively demonstrate the advantages and disadvantages of different technologies from the data [24,25]. Sánchez et al. [26] explored the environmental improvement achieved by completely replacing limestone with marble-waste sludge in the production process of Portland cement. Compared to traditional production processes, this process reduced greenhouse gas emissions by 34%, turbine water usage by 60%, and particulate matter emissions into the atmosphere by 50%. However, in the process of evaluating the feasibility of sludge-based building materials, the focus of research tends to be on whether their mechanical properties meet the standards, and the impact on resources and the environment is usually only qualitatively analyzed, or even directly ignored. Meanwhile, there is little research on the comprehensive evaluation system of properties and environmental benefits of sintered sludge cement (SSC) paste prepared by SSA and activated by different methods.

Herein, this study systematically investigates the effects of SSA treated with different mechanical–thermal activation parameters on the mechanical properties and life cycle assessment of cement paste and conducts a comprehensive evaluation of the activation process. Firstly, SSA is obtained by using different calcination temperatures, calcination durations, and ball-milling times. Then, the SSA is replaced with cement by 30% mass to prepare the SSC paste, and compressive and flexural tests are conducted to achieve strength comparison. Meanwhile, an LCA model for the SSC paste is established by combining the processes of sludge transportation, calcination, and ball milling, in order to conduct resource and environmental impact analysis. Finally, the mechanical strengths and LCA indexes of SSC paste prepared by SSA under different mechanical–thermal activation parameters are comprehensively evaluated through radar charts and the TOPSIS method, providing a theoretical basis for the green and efficient activation of dredged sludge.

## 2. Materials and Methods

### 2.1. Raw Materials

The cementitious materials used in this experiment are ordinary Portland cement (OPC) and dredged sludge, and their chemistry composition is shown in Table 1. The cement used was produced in Fushun, Shenyang. The sludge was directly obtained from the Baiyangdian Lake in Hebei, packaged in barrels and transported to the laboratory. Untreated sludge exhibited a plastic state, poor flowability, high viscosity, with a moisture content of about 40% and organic matter of about 10%, which could not be directly used in experiments and required further treatment. Meanwhile, according to the acetic acid buffer solution method in HJ/T300-2007 [27], there were elements such as As, Cu, Ni, and Zn in the sludge, but each heavy metal element was far below the safety limit specified in the GB 5085.3-2007 standard [28], ensuring its safety. Based on previous research [18], the mineral phases of the dredged sludge used were mainly composed of quartz, albite, muscovite, and clinochlore. The water was directly supplied by ordinary clean water provided by the laboratory.

### 2.2. Mechanical–Thermal Activation of Sludge

After obtaining the sludge from Baiyangdian, it was transported to the laboratory in barrels, without any treatment at this stage. Then, it was spread out on the site (less than 1 m^2^) and dried by natural air drying. The processing time for this stage was 3–4 days. Subsequently, the SM500 × 500 cement test mill was used to crush it for 5 min, thereby obtaining dehydrated sludge ash. According to the experimental settings of 200 °C, 400 °C, 600 °C, 800 °C, and 1000 °C, the dehydrated sludge ash was subjected to high-temperature calcination treatment in batches using the SX2-12-10 box-type resistance furnace made in Hebei, China. After the high-temperature furnace reached the corresponding temperature, the temperature was maintained for 1 or 2 h. After reaching the corresponding time, the power was automatically turned off, and it was taken out and allowed to cool down to room temperature. Finally, the agglomerated sintered sludge was dispersed by ball milling to obtain SSA. The processing time of ball milling was 1 min, 5 min, and 10 min, respectively, followed by 3 min of filtration treatment to obtain three batches of SSA with different ball-milling times.

### 2.3. Mix Proportion

In order to fully utilize the pozzolanic activity of SSA, the SSA was mixed with cement clinker at a replacement rate of 30% to form an SSC paste, designed according to a water/binder of 0.4, as shown in Table 2. Based on previous research [18], the calcination temperature range in this work was controlled within 1000 °C, and was divided into equal parts at intervals of 200 °C, namely 200 °C, 400 °C, 600 °C, 800 °C, and 1000 °C. For the convenience of comparison, the calcination temperature of the sludge obtained by natural drying was recorded as 20 °C. The naming convention for different cement pastes is P-X/Y/Z, where X represents calcination temperature, Y represents calcination duration, and Z represents ball-milling time. For example, P-400/2/1 represents an SSC paste prepared from SSA calcined at 400 °C for 2 h and ball-milled for 1 min.

### 2.4. Preparation of Specimens

**Figure 1:**
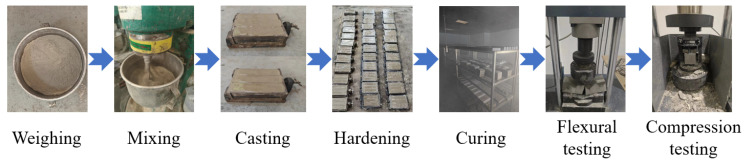
Preparation and testing process of specimens.

Before formally preparing the specimens, rinse the mixing pot with ordinary water in the laboratory, and then invert it on the workbench to dry. Weigh various materials according to the designed mix ratio in Table 2, and put the weighed materials into a mixing pot and stir. After dry mixing for 90 s, add water and continue stirring for 2–3 min until uniform. Then, fill the stirred paste in the mixing pot into a triple mold with a size of 40 × 40 × 160 mm. To avoid any adverse effects that may occur during the vibration process, the filled paste should be slightly higher than the surface of the mold. Place the filled mold in the center of the vibration table, turn on the vibration table and start vibrating for 1–2 min. After the vibration is completed, scrape off the excess paste on the surface of the mold, smooth the surface, cover it with a safety film, and let it sit for 24 h to shape the test piece in the mold. After the test piece is formed, remove the mold and mark it with a black marker on the surface of the test piece. Subsequently, the specimens are moved to a standard curing room for curing under conditions of 20 ± 2 °C and 95% humidity. Finally, the specimens are taken out and subjected to mechanical property testing after being cured for 3 days, 7 days, and 28 days sequentially. Figure 1 shows the above process of specimen preparation and testing.

### 2.5. Test Procedure

#### 2.5.1. Flexural Test

Place the specimen, with dimensions of 40 × 40 × 160 mm, in the three-point bending area of the YAW-300C fully automatic pressure testing machine made in Hebei, China, and adjust the specimen to be in the center position. Set the loading speed to 50 N/s, the gauge length to 100 mm, and take the average of 3 tests on specimens with the same mix proportion.

#### 2.5.2. Compression Test

Place the specimen after the flexural test into the compression fixture of the YAW-300C fully automatic pressure testing machine used for mechanical testing and adjust the specimen to be in the center position on the pressure plate of the fixture. Set the loading speed to 0.5 mm/min and take the average of 3 tests on specimens with the same mix proportion.

#### 2.5.3. Life Cycle Assessment Method

Based on the standard CML-IA baseline V3.05/EU25, this study established a system boundary from cradle to gate, including the transportation, ball milling, calcination, and preparation of cement-based materials for dredged sludge. The entire evaluation process consists of 4 parts: determination of purpose and scope, inventory analysis, impact assessment, and result explanation [29]. The inventory data were obtained from the Ecoinvent database and actual measurements. Considering that the instruments used for treating sludge in this experiment were all electrically driven, this article used an electronic three-phase energy meter to measure the energy consumption data required for sludge treatment, and measured the energy consumption of each processing step 3 times and took the average. Finally, using 1 m^3^ SSC paste as the research unit, the following LCA indicators were calculated using SimaPro 9.4 software: Abiotic Depletion Potential (ADP), Human Toxicity Potential (HTP), Global Warming Potential (GWP), Marine Ecotoxicity Potential (METP), Terrestrial Ecotoxicity Potential (TETP), Acidification Potential (AP), Freshwater Eutrophication Potential (FEP) [30,31].

#### 2.5.4. Radar Chart

Radar chart is a commonly used multi-objective optimization strategy, measured by the size of the area [32,33]. In this study, the maximum mechanical property of different pastes at a specific age was taken as 1 and used as a standard to characterize the mechanical property of each group. For LCA indicators, the minimum value in different pastes was taken as 1, and other values were also characterized. The closer the standardized indicators were to 1, the more they were distributed at the boundaries of the radar chart.

#### 2.5.5. TOPSIS Method

The TOPSIS method, also known as the optimal solution distance method, can be used to analyze and evaluate multiple targets based on different indicator data, and is widely used in scientific project optimization [34,35]. The core idea is to first transform all raw data into a normalized matrix through normalization, and then use the cosine method to determine the optimal and worst solutions in each indicator. Next, evaluate the distance between each indicator in each project corresponding to its optimal and worst solutions, namely the positive ideal solution distance (D+) and the negative ideal solution distance (D−). Finally, by dividing the value of the D− by the sum of the D+ and the D−, the degree of similarity C between the evaluation scheme and the theoretically optimal scheme can be obtained. The larger the C value, the more consistent the evaluated solution is with the theoretically existing optimal solution, indicating that the solution is superior.

## 3. Results and Discussion

### 3.1. Mechanical Property Analysis

#### 3.1.1. Compressive Strength

**Figure 2:**
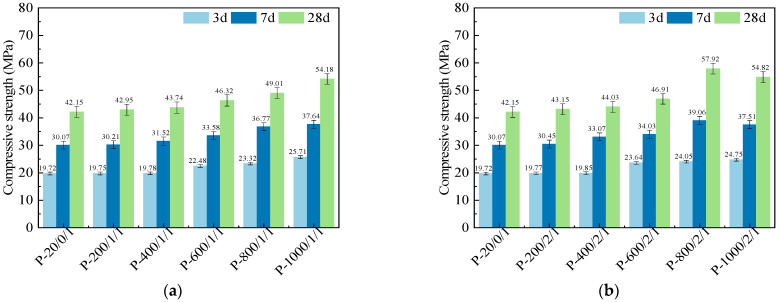
Compressive strengths of SSC pastes prepared by SSA calcined for (**a**) 1 h and (**b**) 2 h.

Figure 2 shows the compressive strengths of the SSC pastes prepared by SSA under different calcination durations. When controlling the ball-milling time to 1 min and the calcination duration to 1 h, the compressive strengths of SSC pastes at 3 days, 7 days, and 28 days continued to increase with the increase in calcination temperature, which was consistent with the research results of Sara et al. [36]. After calcination at 400 °C and below, the increase in compressive strength of SSC paste was minimal. For example, the 3 d compressive strength of P-600/1/1 increased by 19.9% compared to P-20/0/1, while P-400/1/1 only increased by 0.7%. The activation effect of calcination on sludge was only significant after treatment at 600 °C and above. Compared to P-20/0/1, P-1000/1/1 showed an increase in compressive strength of 30.38%, 25.17%, and 28.54% at 3 days, 7 days, and 28 days, respectively. It can be seen that the activation of SSA pozzolanic activity ensured the continuous improvement of SSC paste performance with age. Meanwhile, the activation process of calcination at 1000 °C for 1 h showed the most significant improvement in the 3 d mechanical properties.

When the ball-milling time was controlled at 1 min, and the calcination duration was controlled at 2 h, the 3 d compressive strength increased with the increase in calcination temperature. Although the 3 d compressive strength of P-1000/2/1 was still improved compared to P-800/2/1, its strengths at 7 days and 28 days after sufficient curing were significantly lower than 800 °C. This to some extent indicates that when the temperature exceeded 800 °C, a longer constant temperature time may actually reduce the activation effect of pozzolanic activity, which was related to the microstructure degradation caused by the recrystallization of mineral phases and the appearance of Si-Al spinel [37].

When controlling the ball-milling time for 1 min, compared to the SSC paste prepared by SSA treated with other calcination temperatures and durations, the P-800/2/1 had the highest compressive strength at 7 days and 28 days. Compared to P-20/0/1, P-800/2/1 increased by 29.90% and 37.41% at 7 days and 28 days, respectively. Meanwhile, the growth rates of compressive strength of P-800/2/1 from 3 days to 7 days and 7 days to 28 days were 62.4% and 48.28%, respectively. It can be seen that due to the microstructure reconstruction of SSA under mechanical–thermal activation, the pozzolanic activity was enhanced, and the rate of improvement in the mechanical strength of SSC paste with age had remained at a high level. In addition, except for 1000 °C, the compressive strength of SSC paste increased with the extended duration of sludge calcination.

**Figure 3:**
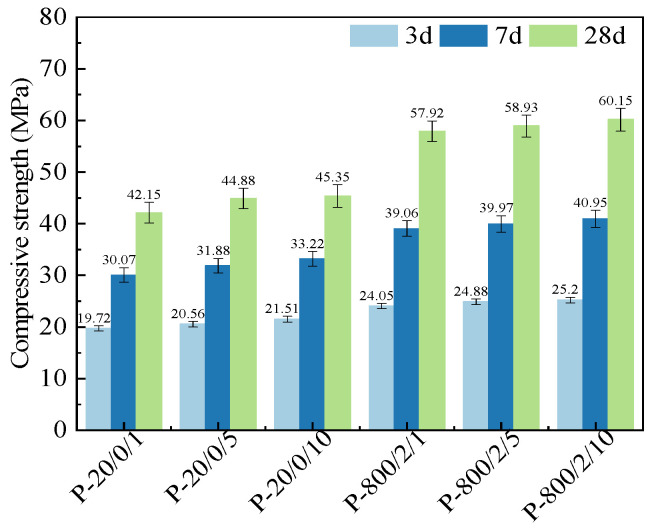
Compressive strengths of SSC pastes prepared by SSA at different ball-milling times.

Figure 3 shows the compressive strengths of SSC pastes prepared by SSA at different ball-milling times. As the ball-milling time was extended from 1 min to 10 min, the compressive strengths were effectively improved in the long term. Compared to P-20/0/1, the increase in compressive strengths of P-20/0/5 and P-20/0/10 at different age groups ranged from 4.26% to 10.48%. Compared to P-800/2/1, the improvement range of P-800/2/5 and P-800/2/10 was only 1.74% to 4.84%. Finally, compared to P-20/0/1, the compressive strengths of P-800/2/10 at 3 days, 7 days, and 28 days increased by 27.79%, 36.18%, and 42.7%, respectively, suggesting a coordinated improvement effect of mechanical–thermal activation.

#### 3.1.2. Flexural Strength

**Figure 4:**
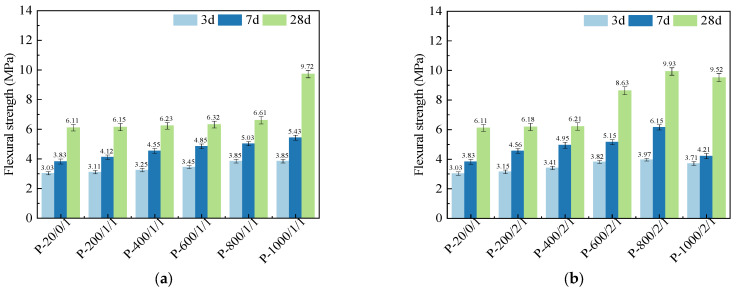
Flexural strengths of SSC pastes prepared by SSA calcined for (**a**) 1 h and (**b**) 2 h.

Figure 4 shows the flexural strengths of SSC pastes prepared by SSA under different calcination durations. When set to a ball-milling time of 1 min, compared to compressive strengths, flexural strengths also increased with the increase in calcination temperature, but its performance required a higher temperature or longer calcination duration to begin to show significant improvement. When the calcination duration was controlled for 1 h, compared to P-20/0/1, the 28 d flexural strengths of P-200/1/1, P-400/1/1, P-600/1/1, P-800/1/1, and P-1000/1/1 increased by 0.65%, 1.93%, 3.44%, 8.18%, and 59.08%, respectively, and the improvement was not significant until 1000 °C. When the calcination duration was controlled for 2 h, it increased by 1.15%, 1.64%, 41.01%, 62.36%, and 55.81% at different calcination temperatures, respectively. Relatively speaking, there was already a significant improvement in flexural strength after 28 days at 600 °C, indicating that longer calcination time was beneficial for reducing the calcination temperature required for sludge activation.

Similar to compressive strengths, longer calcination duration also led to a decrease in flexural strengths at 1000 °C, with a 22.7% decrease at 7 days. However, extending the calcination duration still had a positive effect at 800 °C and below, especially at 600–800 °C. The 28 d flexural strength of P-600/2/1 increased by 37.0% compared to P-600/1/1, and the flexural strength of P-800/2/1 increased by 50.5% compared to P-800/1/1. Similarly, P-800/2/1 had the highest flexural strength at 3 days, 7 days, and 28 days, with increases of 31.02%, 60.57%, and 62.25%, respectively. As the age increased, the improvement effect of flexural strength gradually increased, thanks to the increased pozzolanic activity caused by the restructuring of the SSA microstructure [38].

**Figure 5:**
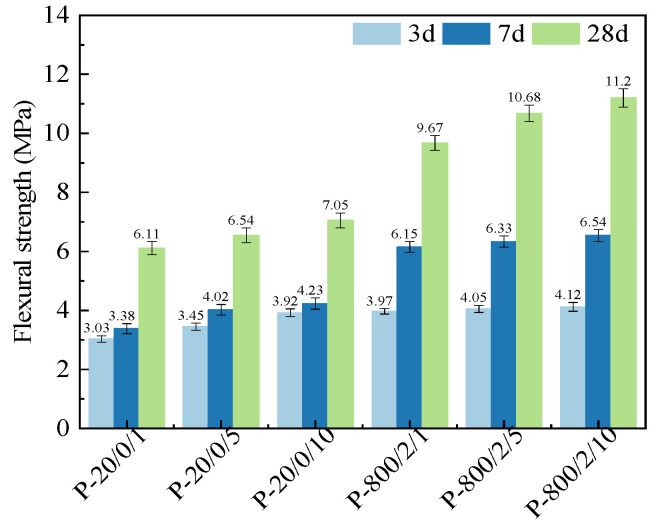
Flexural strengths of SSC pastes prepared by SSA at different ball-milling times.

Figure 5 shows the flexural strengths of SSC pastes prepared by SSA at different ball-milling times. Similar to compressive strength, the flexural strength continued to increase with prolonged ball-milling time. Compared to P-20/0/1, the range of improvement in flexural strengths of P-20/0/5 and P-20/0/10 at different age groups was 7.04% to 29.37%. Compared to P-800/2/1, the improvement range of P-800/2/5 and P-800/2/10 was only 2.02% to 15.82%. It can be seen that the increase in flexural strength was higher than that in compressive strength.

Overall, calcination at 400 °C and below had little effect on the activation of sludge, which may be due to the fact that the temperature range did not reach the temperature required for the activation reactions of various mineral phases in the sludge. When the temperature was between 600 °C and 800 °C, the activation effect improved with the prolongation of calcination duration, which was related to the incomplete reaction of active mineral phases in the sludge caused by short calcination duration. However, a longer holding time at 1000 °C actually led to a decrease in its mechanical properties, possibly due to further reactions of the activated components at 1000 °C, resulting in recrystallization [37]. Meanwhile, as a physical processing method, mechanical ball milling implies that the longer the processing time, the larger the specific surface area of the particles, resulting in a wider range of contact reactions [39]. In addition, the smaller size helped to mix the SSC paste evenly and tightly stack different particles together, thus promoting the improvement in mechanical properties [40].

Therefore, based solely on the comparison and consideration of mechanical properties, it can be concluded that the best choice for the calcination mechanism when mechanically–thermally activating sludge is to calcine at 800 °C for 2 h or at 1000 °C for 1 h, while the time for ball milling sludge is 10 min.

### 3.2. Life Cycle Assessment Model

#### 3.2.1. Determination of Purpose and Scope

**Figure 6:**
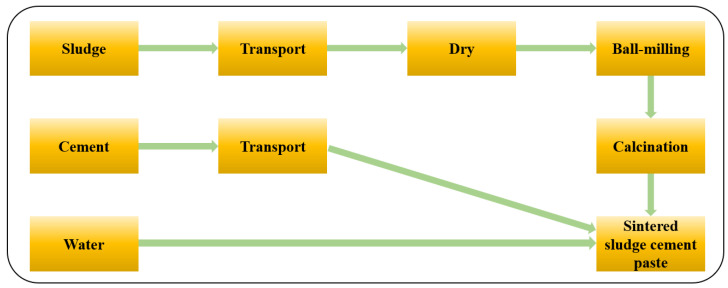
The system boundary.

By constructing an LCA model, it is possible to better quantify the resource and environmental impacts of various sludge treatment conditions and the production of an equal amount of SSC paste. The dehydration and drying of sludge were achieved through long-term natural air drying, which had a low energy consumption and minimal environmental impact. Therefore, the cut-off principle was adopted for the oven treatment stage, and this process was ignored [41,42]. Therefore, the system boundary of the LCA model for preparing SSC paste is shown in Figure 6.

#### 3.2.2. Inventory Analysis

Table 3 and Table 4 record the required electrical energy for sludge treated with different thermal activation parameters and mechanical activation parameters, respectively. From Table 3, it can be observed that, regardless of the temperature, the difference in energy consumption between 1 h of calcination and 2 h of calcination was much smaller than the difference in energy consumption caused by different temperatures. The reason for this phenomenon was that the measured energy consumption started from the start of the heating mode of the high-temperature furnace and ended when the holding time was met, which meant that the energy consumption included the entire heating stage, and the heating stage caused the main energy consumption. These data also indicated that calcination at the same temperature for a longer period of time may be more cost-effective, and the improvement in mechanical properties was greater. Meanwhile, compared to high-temperature calcination, mechanical ball milling generated much less energy consumption.

In this experiment, SSC pastes were prepared with a water/binder ratio of 0.4, and an equal mass of SSA was used instead of cement clinker. Due to the specific gravity of cement clinker and SSA being 3.2 t/m^3^ and 2.0 t/m^3^, respectively, the total mass of SSA, cement clinker, and water required to prepare a 1 m^3^ paste was approximately 1820 kg. Based on the above mix proportion and system boundary, the input situation of each park can be determined as shown in Table 5 and Table 6.

#### 3.2.3. Impact Assessment

The results of relevant resource and environmental indicators are output by modeling and calculating, including ADP, HTP, GWP, METP, TETP, AP, and FEP.

**Figure 7:**
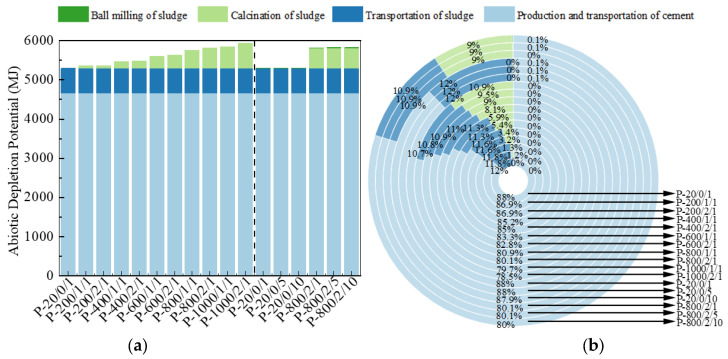
The (**a**) eigenvalue and (**b**) composition ratio of ADP.

The ADP displays the consumption of non-renewable energy sources (fossil fuels) [43], with MJ as the indicator unit, as shown in Figure 7. In the process of preparing SSC paste by replacing 30% cement clinker with SSA, the transportation, ball milling, and calcination of sludge resulted in a maximum fossil fuel consumption of about 20%, which reduced the equivalent amount of cement clinker by about 40% and contributed to sustainable development. Meanwhile, as the calcination temperature and duration of the sludge increased, the consumption of fossil fuels for preparing 1 m^3^ SSC paste gradually increased, but the portion consumed by SSA was still much smaller than that of cement clinker. In addition, the extension of sludge ball-milling time had almost no effect on ADP indicators.

**Figure 8:**
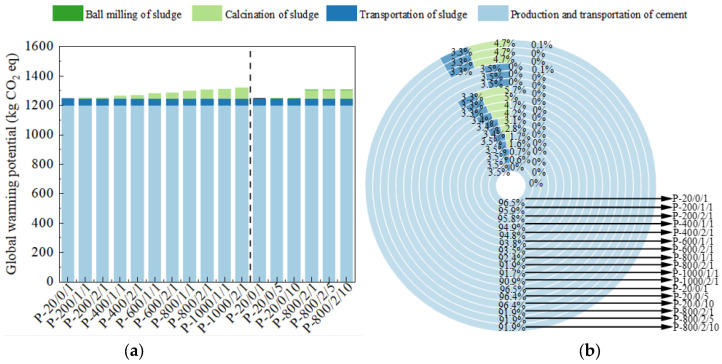
The (**a**) eigenvalue and (**b**) composition ratio of GWP.

The GWP, which expresses the potential for global warming over a 100-year period, is mainly related to greenhouse gas emissions [44]. The unit of this indicator is kg CO_2_ eq, and the calculation results are shown in Figure 8. Compared to ADP, GWP exhibited the same pattern with changes in sludge activation parameters. However, the substitution of SSA had a more significant impact on the reduction of GWP. For example, in P-800/2/10, the 30% SSA generated only accounted for 8.1% of this indicator, which was 77.2% lower than the emission reduction of the same amount of cement clinker, effectively achieving the goal of reducing carbon emissions.

**Figure 9:**
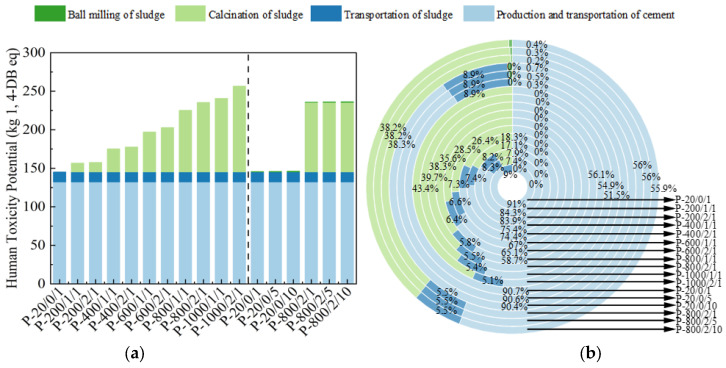
The (**a**) eigenvalue and (**b**) composition ratio of HTP.

The HTP quantifies the harm caused by different emissions to the human body, measured in kg 1,4-DB eq, as shown in Figure 9 [45]. In the process of sludge activation, the influence of high-temperature calcination on HTP accounted for the vast majority. In P-800/2/10, SSA contributed 44.1% of this indicator, which actually exceeded the emissions from cement production. It is speculated that this was due to the electricity selected in the database causing more toxic pollutants during its production process.

**Figure 10:**
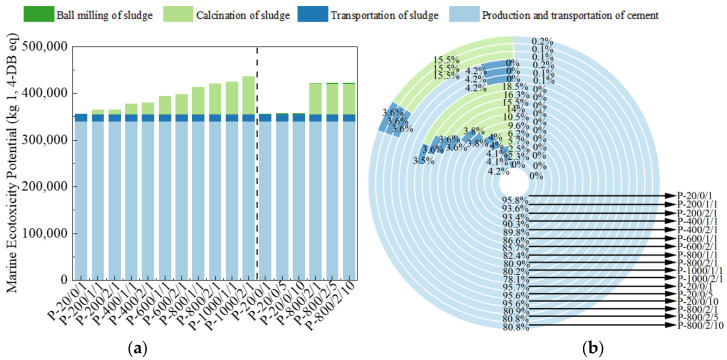
The (**a**) eigenvalue and (**b**) composition ratio of METP.

**Figure 11:**
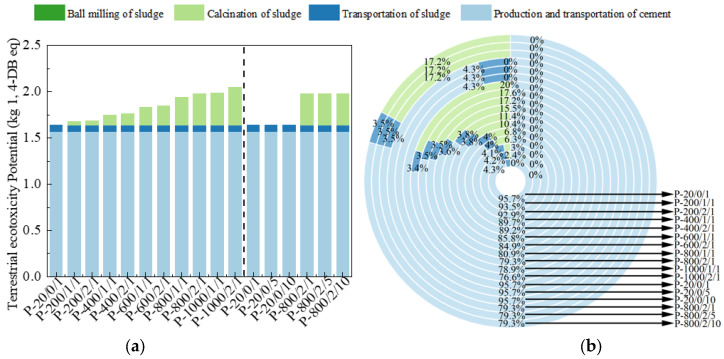
The (**a**) eigenvalue and (**b**) composition ratio of TETP.

The METP and TETP reflect the harm of product lifecycle to ecosystems, measured in kg 1,4-DB eq, as shown in Figure 10 and Figure 11 [46]. The effects of different sludge activation processes on METP and TETP was similar to ADP. For P-800/2/10, the proportion of discharge treated with 30% sludge was 19.2% and 20.7%, respectively, both of which were significantly reduced, reflecting its better ecological and environmental friendliness.

**Figure 12:**
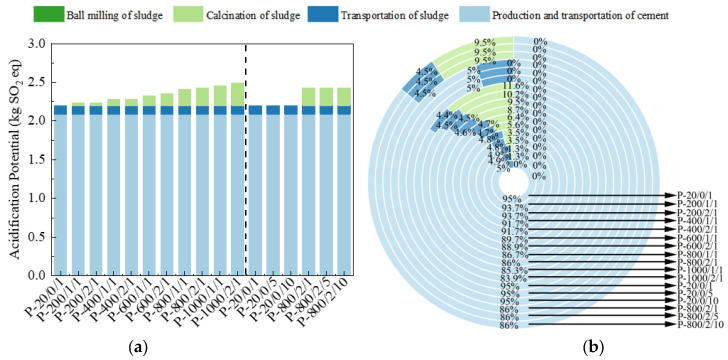
The (**a**) eigenvalue and (**b**) composition ratio of AP.

The AP is calculated based on the mass of SO_2_ emitted per kg of material produced, expressed in kg SO_2_ eq [47]. The calculation results are shown in Figure 12. Similar to other LCA indicators, the addition of SSA significantly reduced the AP, but as the sludge calcination temperature and duration increased, the AP gradually increased. For P-800/2/10, the discharge generated by sludge and its treatment only accounted for 14% of the total discharge, reducing cement usage by 62.0% for the same amount.

**Figure 13:**
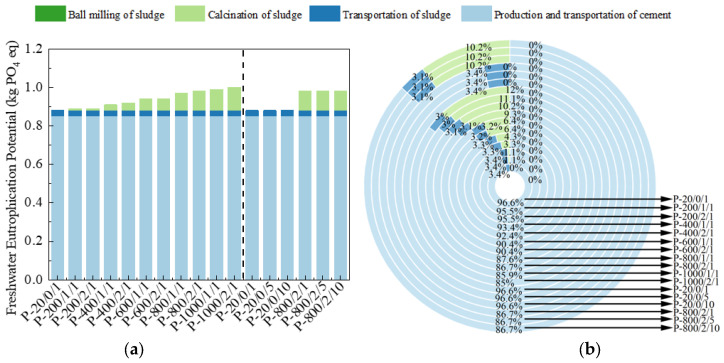
The (**a**) eigenvalue and (**b**) composition ratio of FEP.

The FEP is represented by PO_4_, expressed in kg PO_4_ eq. The calculation results are shown in Figure 13 [48]. For P-800/2/10, the discharge generated by sludge and its treatment only accounted for 14% of FEP, and the equivalent reduction of cement was 65.1%.

Overall, apart from HTP, cement, which accounted for 70% of raw material consumption, generated 80% of emissions in other LCA indicators, with the highest proportion of global warming potential (GWP) indicators reaching as much as 91.9%. This was consistent with the originally assumed situation, and it was speculated that the main reason was that the temperature for processing sludge, even at a maximum of 1000 °C, was much lower than the 1450 °C required for cement clinker preparation, which required much lower fossil fuels and energy than the latter.

#### 3.2.4. Result Explanation

Based on the information obtained from modeling, sensitivity analysis can be conducted at each stage of SSC paste preparation to identify the most optimized stage [49]. In this project, a road sensitivity analysis was used to achieve this. This method compares each parameter by setting a percentage change in the listed results and analyzing the corresponding proportions that need to be changed. The smaller the required change ratio of a parameter, the higher its sensitivity. According to the data in Figure 7, Figure 8, Figure 9, Figure 10, Figure 11, Figure 12 and Figure 13, it can be observed that ADP, GWP, METP, AP, and FEP have the highest environmental impact index values and no intersection with each other. Therefore, these five indicators were selected as sensitivity analysis and subsequent main research objects in this project. In order to ensure that all links are reflected in the same model, the SSC paste prepared by SSA calcined at 800 °C for 2 h and milled for 10 min was used as the object for research. Assuming that the total amount changes by 10%, its sensitivity in all aspects is shown in Table 7, Table 8, Table 9, Table 10 and Table 11.

According to Table 7, Table 8, Table 9, Table 10 and Table 11, it can be found that the most sensitive indicators were the production and transportation of cement, while the impact caused by ball milling was relatively small. Therefore, in practical applications, if the proportion of SSA replacing cement can be further increased, it can greatly reduce the impact on related resources and the environment. Similarly, if the mechanical properties can be further improved through continuous ball milling operations, the ball-milling time can be appropriately extended.

For P-800/2/10, significant reductions in emissions caused by the complete use of cement can be achieved in all five selected aspects. The impact on global warming is particularly evident, accounting for only 8.05% of the total emissions with 30% SSA. However, in other resource and environmental impacts with relatively small values, some indicators, such as HTP, reflected higher emissions caused by the mixing of SSA. However, their values were relatively small and, compared to other reduced emissions, their impact could be acceptable.

### 3.3. Multi-Criteria Evaluation

**Figure 14:**
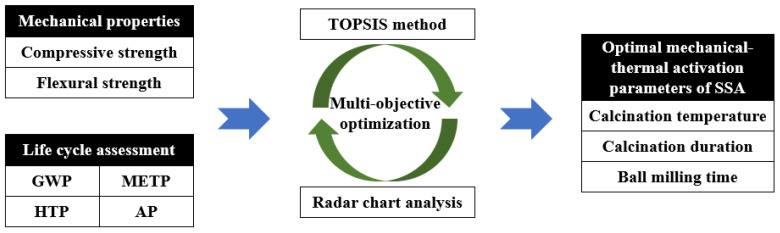
Analysis flowchart.

Based on the above discussion, this study has measured the specific values of the mechanical properties and resource environmental impact indicators of the corresponding cementitious material, and the optimal treatment process can be determined separately from a certain aspect. However, it is still difficult to achieve a balance between mechanical properties and environmental benefits. This section adopts radar chart analysis and the TOPSIS method for comprehensive evaluation, and selects the optimal sludge activation process in this experiment. The analysis process executed above is shown in Figure 14.

#### 3.3.1. Radar Chart Analysis

**Figure 15:**
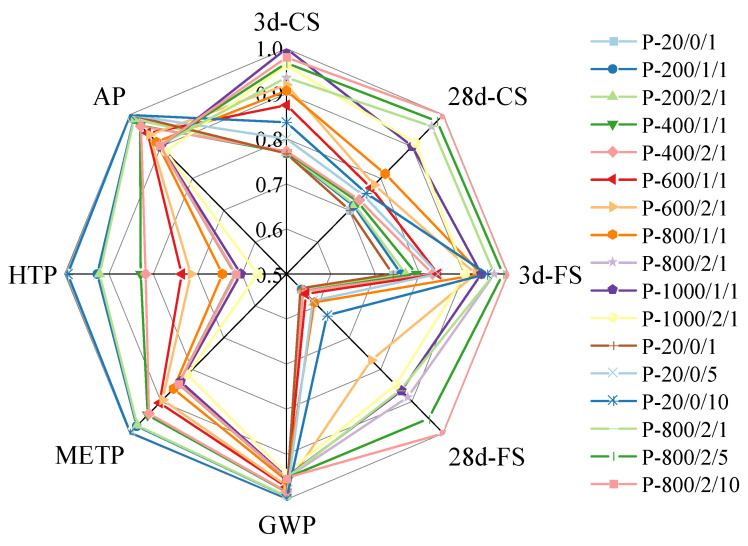
Radar chart.

Considering the mechanical properties and environmental benefits of the SSC paste, this article set the mechanical evaluation indicators as compressive strength and flexural strength. Due to the non-biological depletion of fossil fuels, which was often closely related to greenhouse gas emissions, only the GWP indicator was selected as the optimized data for the two. In terms of environmental benefits, emissions from four aspects were selected as evaluation indicators: GWP, METP, HTP, and AP. The scores of SSC pastes prepared by SSA under different activation modes are shown in Table 12, and the corresponding radar chart is shown in Figure 15.

According to Figure 15, the radar chart area under different activation processes was calculated. It is found that P-800/2/10 obtained the best comprehensive benefit, which was reflected in the radar chart as occupying the maximum score area of 7.29. From a temperature perspective alone, both P-1000/1/1 and P-1000/2/1 were lower than P-800/2/1, due to the latter requiring lower energy consumption and resulting in less reduction in environmental benefits. For the experimental groups at 200 °C and 400 °C, the improvement in mechanical properties was not significant, but it caused more environmental burden, resulting in scores even lower than those of the untreated group. In terms of ball-milling duration, ball milling within 10 min always improved the mechanical properties of the SSC paste. As a mechanical treatment method, the energy consumption required for the activation process of ball milling and crushing was much lower than that of calcination, resulting in less reduction in environmental benefits. Its mechanical improvement compensated for the negative environmental impact caused by longer ball-milling duration.

#### 3.3.2. TOPSIS Analysis

(1) Calculation of indicator weights

The TOPSIS method was used to comprehensively evaluate the benefits of various activation processes, and it is necessary to confirm the weights of each indicator in the evaluation system. In this article, the entropy weight method was used to achieve this.

Using the same mechanical performance indicators and resource and environmental impact indicators as radar chart analysis, the weight calculations were performed to obtain the weights of each indicator shown in Table 13.

(2) Comprehensive score calculation

According to the TOPSIS algorithm, the corresponding comprehensive score of the distance between the superior and inferior solutions was calculated and sorted, as shown in Table 14.

According to the TOPSIS method, the optimal solution was still to calcine at 800 °C for 2 h and ball mill for 10 min. This result was consistent with previous findings calculated using the radar chart, further demonstrating the reliability of the analysis conclusions for the optimal solution. The analysis suggests that due to the higher weight of mechanical indicators in the comprehensive benefit evaluation, the better mechanical properties of SSC paste under this treatment method had earned it a higher rating. However, it should also be noted that even though the mechanical properties of P-600/2/1, such as compressive strength, were significantly lower than those of P-1000/2/1, and the latter was 16.8% higher than the former, its comprehensive benefits were still greater than those of the latter. This was due to the significant increase in emissions caused by the increase in calcination temperature, which reduced its overall benefits, indicating that the environmental benefits played an indispensable role in comprehensive evaluation.

## 4. Conclusions

This study systematically explored the influence of SSA under different mechanical–thermal activation parameters on the mechanical properties and LCA index of cement paste. Subsequently, based on the multi-objective evaluation method, the optimal combination of activation parameters was determined.

(1) At temperatures below 1000 °C, higher calcination temperature and longer calcination duration were accompanied by corresponding improvements in the mechanical properties of the SSC paste. However, this effect was not prominent at temperatures below 400 °C. The longer holding time at 1000 °C actually resulted in a decrease in its mechanical properties, which was related to the recrystallization of activated mineral phases. In addition, the effect of ball milling duration on the strength of the SSC paste showed an increasing relationship, especially in terms of flexural strength.
(2) Among the various stages of the LCA model, cement production and transportation had the highest sensitivity. This meant that reducing the amount of cement clinker used was the most effective solution to alleviate the impact on resources and the environment. The temperature increase during sludge activation had a significant impact on energy consumption, greenhouse gases, and other resources and environments. With the addition of SSA, the indicators of ADP, GWP, METP, TETP, AP, and FEP were effectively reduced. Particularly, GWP, which accounted for 30% of SSA from transportation to processing, only generated 8.1% of total greenhouse gas emissions.
(3) Based on the radar chart and the TOPSIS method, the sludge activation method involving calcination at 800 °C for 2 h and ball milling for 10 min achieved the highest comprehensive benefit score in terms of performance and environmental indicators.

Further research will focus on the synergistic use of different SCMs and SSA in cement paste. In order to achieve appropriate levels of Ca, Si, and Al in the cementitious system, fly ash, slag, metakaolin, and limestone will be given special consideration. This can not only further reduce the dosage of cement clinker, but also improve performance by optimizing the compatibility of the cementitious components.

## Figures and Tables

**Table 1:** Chemical composition of cementitious materials.

Material	SiO_2_	Al_2_O_3_	Fe_2_O_3_	CaO	MgO	SO_3_	f-CaO	LOI
OPC	22.95	8.90	3.30	57.36	2.36	2.75	0.88	1.50
Sludge	57.61	19.01	7.48	2.51	3.62	0.36	-	9.60

**Table 2:** The activation process of SSA and its corresponding SSC paste mix ratio.

Number	Cement (%)	SSA				Water (%)
		Calcination Temperature (°C)	Calcination Duration (Hour)	Ball-Milling Time (Minute)	Content (%)	
P-20/0/1	70	20 (Blank control)	0	1	30	40
P-200/1/1		200	1	1		
P-200/2/1		200	2	1		
P-400/1/1		400	1	1		
P-400/2/1		400	2	1		
P-600/1/1		600	1	1		
P-600/2/1		600	2	1		
P-800/1/1		800	1	1		
P-800/2/1		800	2	1		
P-1000/1/1		1000	1	1		
P-1000/2/1		1000	2	1		
P-20/0/5		20	0	5		
P-20/0/10		20	0	10		
P-800/2/5		800	2	5		
P-800/2/10		800	2	10		

**Table 3:** Energy consumption generated by high-temperature calcination of sludge.

Calcination Temperature (°C)	Calcination Duration (Hour)	Electricity Consumption (kW·h)
200	1	3.14
200	2	3.38
400	1	8.17
400	2	8.83
600	1	14.16
600	2	15.73
800	1	21.81
800	2	24.57
1000	1	26.02
1000	2	30.34

**Table 4:** Energy consumption generated by mechanical ball milling of sludge.

Mechanical Activation	Electricity Consumption (kW·h)
Crush for 5 min	0.08
Ball milling for 1 min	0.01
Ball milling for 5 min	0.08
Ball milling for 10 min	0.15
Filter for 3 min	0.04

**Table 5:** Raw material input list for preparing 1 m^3^ SSC paste.

Material	Cement	SSA	Water
Content (kg)	910	390	520

**Table 6:** Input list of transportation distance for preparing SSC paste.

Material	Cement	Sludge	Water
Transportation distance (km)	13	150	0

**Table 7:** Sensitivity analysis of ADP.

LCA Index	Total	Production and Transportation of Cement	Transportation of Sludge	Ball Milling of Sludge	Calcination of SSA
ADP	1.85 × 10^−10^	1.48 × 10^−10^	2.02 × 10^−11^	1.83 × 10^−13^	1.66 × 10^−11^
10% sensitivity	1.85 × 10^−11^	12.5%	91.6%	10,109.3%	111.4%

**Table 8:** Sensitivity analysis of GWP.

LCA Index	Total	Production and Transportation of Cement	Transportation of Sludge	Ball Milling of Sludge	Calcination of SSA
GWP	2.60 × 10^−10^	2.39 × 10^−10^	8.70 × 10^−12^	1.34 × 10^−13^	1.22 × 10^−11^
10% sensitivity	2.60 × 10^−11^	10.9%	298.9%	19,403.0%	213.1%

**Table 9:** Sensitivity analysis of METP.

LCA Index	Total	Production and Transportation of Cement	Transportation of Sludge	Ball Milling of Sludge	Calcination of SSA
METP	3.61 × 10^−9^	2.92 × 10^−9^	1.29 × 10^−10^	6.14 × 10^−12^	5.58 × 10^−10^
10% sensitivity	3.61 × 10^−10^	12.7%	279.8%	5879.5%	64.7%

**Table 10:** Sensitivity analysis of AP.

LCA Index	Total	Production and Transportation of Cement	Transportation of Sludge	Ball Milling of Sludge	Calcination of SSA
AP	8.63 × 10^−11^	7.42 × 10^−11^	3.75 × 10^−12^	9.07 × 10^−14^	8.26 × 10^−12^
10% sensitivity	8.63 × 10^−12^	11.6%	230.1%	9514.9%	104.5%

**Table 11:** Sensitivity analysis of FEP.

LCA Index	Total	Production and Transportation of Cement	Transportation of Sludge	Ball Milling of Sludge	Calcination of SSA
FEP	7.37 × 10^−11^	6.41 × 10^−11^	2.00 × 10^−12^	8.27 × 10^−14^	7.53 × 10^−12^
10% sensitivity	7.37 × 10^−12^	11.5%	368.5%	8911.7%	97.9%

**Table 12:** The scores of SSC pastes prepared by SSA under different activation modes.

	3 d Compressive Strength	28 d Compressive Strength	3 d Flexural Strength	28 d Flexural Strength	Global Warming Potential	Marine Ecotoxicity Potential	Human Toxicity Potential	Acidification Potential	Total
P-20/0/1	0.77	0.70	0.74	0.55	1.00	1.00	1.00	1.00	6.75
P-200/1/1	0.77	0.71	0.76	0.55	0.99	0.98	0.93	0.99	6.67
P-200/2/1	0.77	0.72	0.77	0.55	0.99	0.98	0.92	0.99	6.68
P-400/1/1	0.77	0.73	0.79	0.55	0.98	0.94	0.83	0.97	6.56
P-400/2/1	0.77	0.73	0.83	0.55	0.98	0.94	0.82	0.96	6.59
P-600/1/1	0.88	0.77	0.84	0.56	0.97	0.90	0.74	0.94	6.60
P-600/2/1	0.92	0.78	0.93	0.77	0.97	0.90	0.72	0.94	6.91
P-800/1/1	0.91	0.82	0.94	0.59	0.96	0.86	0.64	0.91	6.63
P-800/2/1	0.94	0.96	0.97	0.89	0.95	0.84	0.62	0.90	7.07
P-1000/1/1	1.00	0.90	0.94	0.87	0.95	0.84	0.60	0.90	7.00
P-1000/2/1	0.96	0.91	0.90	0.85	0.94	0.82	0.57	0.88	6.83
P-20/0/1	0.77	0.70	0.74	0.55	1.00	1.00	1.00	1.00	6.75
P-20/0/5	0.80	0.75	0.84	0.58	1.00	1.00	1.00	1.00	6.96
P-20/0/10	0.84	0.75	0.95	0.63	1.00	1.00	0.99	1.00	7.16
P-800/2/1	0.94	0.96	0.97	0.87	0.95	0.84	0.62	0.90	7.07
P-800/2/5	0.97	0.98	0.99	0.95	0.95	0.84	0.62	0.90	7.20
P-800/2/10	0.98	1.00	1.00	1.00	0.95	0.84	0.61	0.90	7.29

**Table 13:** Calculation results based on entropy weight method.

Indicator	Information Entropy Value	Information Utility Value	Weight (%)
3d-CS	0.83	0.17	17.21
28d-CS	0.84	0.16	16.16
3d-FS	0.90	0.10	9.80
28d-FS	0.77	0.23	22.45
GWP	0.91	0.09	8.60
METP	0.91	0.09	8.60
HTP	0.91	0.09	8.60
AP	0.91	0.09	8.60

Note: Information entropy value is used to measure the uncertainty of a random variable, and it increases as the uncertainty increases. Information utility value is used to measure the importance of indicators and determine the weight of each indicator.

**Table 14:** Calculation results of the superior inferior solution distance method.

Number	Positive Ideal Solution Distance (D+)	Negative Ideal Solution Distance (D−)	Comprehensive Score Index	Rank
P-20/0/1	0.81	0.59	0.42	9
P-200/1/1	0.79	0.53	0.40	11
P-200/2/1	0.78	0.52	0.40	10
P-400/1/1	0.78	0.43	0.36	15
P-400/2/1	0.76	0.43	0.36	14
P-600/1/1	0.68	0.40	0.37	13
P-600/2/1	0.51	0.52	0.50	6
P-800/1/1	0.67	0.41	0.38	12
P-800/2/1	0.51	0.65	0.56	3
P-1000/1/1	0.54	0.65	0.54	4
P-1000/2/1	0.63	0.58	0.48	7
P-20/0/1	0.81	0.59	0.42	9
P-20/0/5	0.68	0.60	0.47	8
P-20/0/10	0.59	0.66	0.53	5
P-800/2/1	0.51	0.64	0.55	3
P-800/2/5	0.49	0.74	0.60	2
P-800/2/10	0.48	0.80	0.62	1

Note: The positive ideal solution distance (D+) is the Euclidean distance between the decision solution and the positive ideal solution, used to measure the degree to which the decision solution approaches the optimal state. The negative ideal solution distance (D−) is the Euclidean distance between the decision solution and the negative ideal solution, used to measure the degree to which the solution is far from the worst state. The comprehensive score index is the degree to which a decision solution approaches the positive ideal solution, defined as the ratio of the negative ideal solution distance to the total distance (the sum of the positive and negative ideal solution distances).

## Data Availability

The original contributions presented in the study are included in the article, further inquiries can be directed to the corresponding author/s.
